# Home-Based Monitoring of Treatment-Related Adverse Events during Drug-Resistant Tuberculosis Treatment, India, 2020–2024

**DOI:** 10.3201/eid3203.251893

**Published:** 2026-03

**Authors:** Minal Ahson, Daksha Shah, Sampada Bhide, Rajesh Deshmukh, Jonathan P. Smith, Smita Waghmare, Satish Kaipilyawar, Varsha Puri, Dilip K. Khetade, Vijay Yeldandi, Anand Date, Patrick K. Moonan, Christine S. Ho

**Affiliations:** Centers for Disease Control and Prevention, Atlanta, Georgia, USA (M. Ahson, J.P. Smith, A. Date, P.K. Moonan, C.S. Ho); Brihanmumbai Municipal Corporation, Mumbai, India (D. Shah, V. Puri, D.K. Khetade); Society for Health Allied Research and Education, Hyderabad, India (S. Bhide, S. Waghmare, S. Kaipilyawar, V. Yeldandi); US Centers for Disease Control and Prevention, Mumbai (R. Deshmukh)

**Keywords:** Tuberculosis and other mycobacteria, TB, Mycobacterium tuberculosis, bacteria, respiratory infections, antimicrobial resistance, multidrug-resistant bacteria, multidrug-resistant tuberculosis, extensively drug-resistant tuberculosis, drug-related adverse reactions, pharmacovigilance, community-based care, treatment adherence, India

## Abstract

We investigated home-based outreach and point-of-care diagnostic tools for monitoring adverse events among persons treated for drug-resistant tuberculosis in Dharavi, India. Of 974 persons treated, 518 (53%) reported 1,410 adverse events, 126/477 (26%) required regimen change, and 83% of patients completed therapy. Home-based adverse event monitoring can help improve tuberculosis treatment adherence.

India contributes 25% of the tuberculosis (TB) burden and 32% of drug-resistant TB (DR TB) cases worldwide (*1*). DR TB in Mumbai ranks among the highest globally (*2*). Within Mumbai, Dharavi is one of the world’s largest informal settlements, housing >1 million persons in 0.8 square miles and supporting ≈15,000 small factories employing >250,000 persons (*3*).

Completing DR TB treatment in Dharavi remains challenging because many patients are migrants who lack stable family support during treatment (*4*). DR TB treatment regimens are prolonged and rely on second-line drugs, which are more toxic than those used for drug-susceptible TB. Drug-related adverse events are common during DR TB treatment and complicate clinical management (*5*). Consequently, DR TB is associated with higher relapse and mortality rates (*1*). 

The development of shorter, more tolerable DR TB regimens offers opportunities to improve and decentralize care. However, data on drug-specific adverse events in community-based programs remain limited despite global recommendations for active monitoring (*6*). We evaluated a home-based adverse event monitoring system implemented in Dharavi to assess patient adherence to treatment regimens and feasibility of such programs in resource-limited settings.

## The Study

The End DRTB in Dharavi project included a series of programmatic interventions aimed at improving treatment adherence and outcomes among patients with DR TB in that area (*7*). In brief, all adult (>18 years of age) DR TB patients within Dharavi who initiated government-supported treatment during December 2020–June 2022 were eligible for inclusion. The project prospectively enrolled TB patients who initiated treatment for multiple phenotypic drug-resistance patterns, including monoresistant (i.e., resistance to 1 first-line drug), polyresistant (i.e., resistance >2 first-line drugs but not to both isoniazid and rifampin), multidrug-resistant (MDR; i.e., resistance to at least isoniazid and rifampin), and extensively drug-resistant (XDR; i.e., MDR and resistance to any fluoroquinolone and a second-line injectable drug [SLID]). All participants received drug-susceptibility guided treatment, according to national guidelines (*8*). Urban Health Centre (UHC) Dharavi provided baseline testing to assess toxicity, including audiometry, electrocardiogram (ECG), visual acuity and Snellen tests, and comprehensive metabolic blood panels for pretreatment evaluation. We provided point-of-care, tablet-based audiometry (Shoebox, https://www.shoebox.md) and electrocardiogram (SmartHeart, https://www.getsmartheart.com) tests to expedite pretreatment evaluation and reduce travel requirements for testing. Trained field coordinators subsequently recorded clinical adverse events during monthly home visits by using a standardized screening and referral questionnaire (Appendix Figure), audiometry, and electrocardiogram testing, following a previously described predetermined schedule (*8*). 

We categorized adverse events as mild, moderate, or severe. Severe adverse events resulted in hospitalization, persistent disability, a life-threatening condition, or death and required clinical intervention to prevent or manage those outcomes (*7*). Moderate adverse events required clinical intervention, symptomatic treatment, or treatment modification but not hospitalization, and mild adverse events did not require clinical intervention or treatment modification. We referred patients reporting any adverse event to the nearest medical officer for evaluation, per standard guidelines (*8*), and referred patients with abnormal audiometry or ECG findings or any severe adverse event to a tertiary care facility or the UHC chest physician for further management and any necessary treatment regimen modifications.

Our primary outcomes were occurrence and timing of any clinically relevant adverse event or abnormality (audiometric, cardiac, optic, or metabolic) during DR TB treatment. We excluded from analysis adverse events that were reported at baseline, before the medication regimen began. We counted adverse events reported at consecutive visits as a single event unless severity increased and considered nonconsecutive episodes separate events. Because hearing loss is generally irreversible, we classified it as a nonrecurring event but recorded any worsening (*9*).

We used frequencies and proportions to describe new adverse events during treatment, stratified by regimen and antimicrobial drugs. We assessed group differences by using χ^2^ or Monte Carlo simulation methods, as appropriate. We analyzed time to first event by using cumulative incidence function with Gray’s method, accounting for competing risks, including death, loss to follow-up, treatment discontinuation, and transfers out of the service area. We estimated subdistribution hazard ratios and 95% CIs by using the Fine-Gray model with weighted Cox regression. This activity was approved by the Brihanmumbai Mumbai Corporation and the US Centers for Disease Control and Prevention and was determined to be nonresearch.

Among 974 DR TB patients, 880 (90%) started MDR TB treatment, 51 (5%) started monoresistant TB treatment, 38 (4%) started XDR TB treatment, and 5 (<1%) started polyresistant TB regimens (Appendix Table). Overall, 518 (53%) patients reported a total of 1,410 discrete adverse events, the most frequent of which were gastrointestinal (22%), neurologic (21%), and musculoskeletal (12%) events (Table). Most (96%, n = 1,359) events were mild or moderate; 38 (7%) patients experienced 51 (4%) severe events. Participants receiving MDR TB regimens were more likely (55%) to report >1 adverse event than those who received monoresistant or polyresistant (41%) or XDR TB regimens (40%) (p = 0.04), none of which included SLIDs. Participants on a regimen containing SLIDs were almost 4 times as likely to report an adverse event compared with those not using SLIDs (hazard ratio 3.87 [95% CI 3.14–4.77]) (Figure). Of patients who received SLIDs, 206 (89%) completed treatment. Overall, 805 (83%) patients completed TB treatment.

**Table:** Frequency, severity, and treatment modification of reported adverse events during home-based monitoring of drug-resistant tuberculosis treatment, Dharavi, Mumbai, India, 2020‒June 2024*

Adverse event†	Total	Severity			Required treatment modification
		Mild	Moderate	Severe	
Neurologic	292 (21)	86 (29)	203 (70)	3 (1)	52 (18)
Tingling, pain, or numbness in hands or feet	148 (11)	48 (32)	99 (67)	1 (1)	34 (23)
Visual disturbances	54 (4)	15 (28)	39 (72)	0	12 (22)
Headache	34 (3)	7 (21)	26 (76)	1 (3)	3 (9)
Dizziness	27 (2)	8 (30)	19 (70)	0	0
Hearing loss	16 (1)	1 (6)	15 (94)	0	1 (6)
Ringing in ears	12 (1)	7 (58)	5 (42)	0	2 (17)
Seizure	1 (<1)	0	0	1 (100)	0
Gastrointestinal	309 (22)	123 (40)	175 (57)	11 (4)	12 (4)
Nausea, vomiting	163 (12)	79 (48)	80 (49)	4 (3)	6 (4)
Loss of appetite	68 (5)	24 (35)	43 (63)	1 (2)	3 (4)
Abdominal pain	51 (4)	13 (25)	33 (65)	5 (10)	2 (4)
Diarrhea	13 (1)	3 (23)	10 (77)	0	1 (8)
Constipation	8 (<1)	2 (25)	6 (75)	0	0
Difficulty urinating	4 (<1)	1 (25)	2 (50)	1 (25)	0
Yellowish discoloration of skin and eyes	2 (<1)	1 (50)	1 (50)	0	0
Musculoskeletal	174 (12)	66 (38)	108 (62)	0	23 (13)
Joint pain	168 (12)	65 (39)	103 (61)	0	22 (13)
Neck, face swelling	6 (<1)	1 (17)	5 (83)	0	1 (17)
Behavioral	48 (4)	18 (38)	26 (54)	4 (8)	10 (21)
Psychosis	10 (1)	6 (60)	4 (40)	0	2 (20)
Changes in behavior	18 (1)	6 (33)	11 (61)	1 (6)	5 (28)
Sleep disturbances	8 (<1)	3 (38)	5 (62)	0	1 (13)
Depression or suicidal ideation	12 (1)	3 (25)	6 (50)	3 (25)	2 (17)
Dermatologic	37 (3)	21 (57)	16 (43)	0	3 (8)
Skin rash or itching	37 (3)	21 (57)	16 (43)	0	3 (8)
Cardiologic	99 (7)	28 (28)	66 (67)	5 (5)	5 (5)
Abnormal electrocardiogram	39 (3)	20 (51)	19 (49)	0	0
Fatigue	38 (3)	4 (11)	31 (82)	3 (8)	3 (8)
Palpitations	22 (2)	4 (18)	16 (73)	2 (9)	2 (9)
Other, unspecified	451 (32)	139 (31)	284 (63)	28 (6)	21 (5)
Total	1,410	481 (34)	878 (62)	51 (4)	126 (9)

*Values are no. (%). †Patients could experience multiple events.

**Figure:**
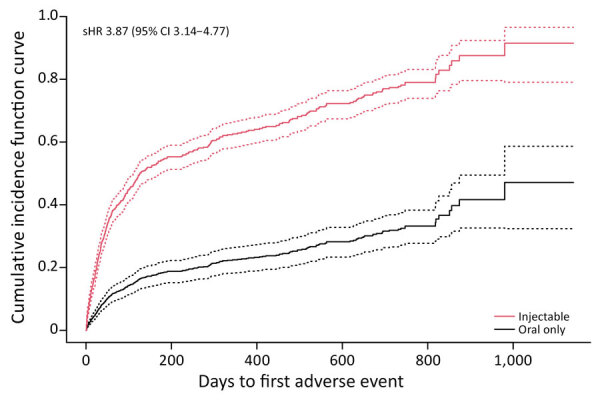
Cumulative incidence function curves of time to first clinical adverse events in a study of home-based monitoring of treatment-related adverse events during drug-resistant tuberculosis treatment, Dharavi, Mumbai, India, 2020‒2024. Graphs compare reactions between patients with injectable versus noninjectable treatment regimens among patients with drug-resistant TB. Solid lines indicate medians; dotted lines indicate 95% CIs. sHR, subdistribution hazard ratio.

Treatment modification data were available for 477 (92%) patients who reported an adverse event. Among those, 126 (26%) reported >1 regimen change attributable to their event, and 57 (45%) regimen changes occurred at first adverse event. Two patients developed clinically apparent jaundice, corroborated by elevated hepatic transaminase and bilirubin levels; both required treatment regimen modification. Thirty-nine (3%) patients had abnormal ECG findings and were referred to UHC chest physicians for further evaluation; none required treatment regimen modification. Among 233 patients receiving SLIDs, 219 (94%) had baseline audiometric screening, and 173 (79%) had abnormal follow-up results. Of those, 100 (58%) were evaluated by an otolaryngologist or audiology subspecialist, and 14 (14%) discontinued SLIDs after assessment. Regimen changes enabled patients to continue treatment safely and did not necessarily result in treatment interruption or discontinuation. Our findings aligned with results from an individual patient data meta-analysis on MDR TB, which demonstrated poorer outcomes among patients treated with kanamycin or capreomycin SLIDs (*10*).

## Conclusions

This home-based monitoring model coincided with sustained care engagement and adherence; 83% of patients completed therapy in Dharavi, compared with ≈1 in 3 before implementation of this model (*7*). Even among patients on SLID-containing regimens, the group with the most adverse events, 89% completed treatment. Although not a causal evaluation, our findings suggest that routine adverse event monitoring, detection, and timely management supported improved treatment adherence and completion. SLIDs have been downgraded in national guidelines (*7*), but several factors highlight the need to reassess their role in TB treatment. Those factors include rising bedaquiline resistance; challenges accessing bedaquiline, pretomanid, linezolid, and moxifloxacin–based regimens; frequent linezolid toxicity; and limited access to drug-susceptibility testing (*11*,*12*). Given their high resistance threshold, SLIDs might remain useful when judiciously applied. Emerging evidence that 2 early high-dose amikacin doses can reduce initial resistance without added short-term toxicity (*13*) supports a limited transitional role for SLIDs within evolving all-oral, patient-centered treatment models.

In conclusion, sustained investments in local capacity and integration of patient-centered monitoring within national TB programs are essential to achieving global End TB targets (*14*). We found that active home-based monitoring for adverse events and use of point-of-care diagnostic tools were feasible and effective in this high-burden, resource-constrained setting. That approach improved early detection and management of drug-related toxicities and sustained engagement in care and adherence among patients with DR TB. Implementation of decentralized adverse event surveillance and mobile health technologies can strengthen pharmacovigilance and improve treatment outcomes in similar high-density, informal settlements. 

Appendix Additional information on home-based monitoring of treatment-related adverse events during drug-resistant tuberculosis treatment, India, 2020–2024.
