# Supplementary material for: Home-Based Monitoring of Treatment-Related Adverse Events during Drug-Resistant Tuberculosis Treatment, India, 2020–2024
Source: Emerg Infect Dis. 2026 Mar;32(3):419–23. doi: 10.3201/eid3203.251893 (PMC13016008; doi:10.3201/eid3203.251893)
Supplement: Appendix — Additional information on home-based monitoring of treatment-related adverse events during drug-resistant tuberculosis treatment, India, 2020–2024. [file 25-1893-Techapp-s1.pdf]

Article DOI: https://doi.org/10.3201/eid3203.251893

EID cannot ensure accessibility for supplementary materials supplied by authors. Readers who have difficulty accessing supplementary content should contact the authors for assistance.

Home-Based Monitoring of Treatment-Related Adverse Events during Drug-Resistant Tuberculosis Treatment, India, 2020–2024

Appendix

Appendix Table. Frequency of drug-resistant TB patients by National Tuberculosis Elimination Program-defined treatment regimens in Dharavi, Mumbai, December 2020–June 2022

Regimen	TB Type	No. (%)	LFX	R	Z	E	BDQ	MFX	ETO	CFZ	H	LZD	CS	AM	KM	PAS	CM
All oral H-mono/poly-resistance DR TB regimen	DR	51 (5.2%)	Y	Y	Y	Y	–	–	–	–	–	–	–	–	–	–	–
Shorter MDR TB regimen	MDR	151 (15.5%)	Y	–	Y	Y	Y	Y	Y	Y	Y	–	–	–	–	–	–
All oral longer MDR TB regimen	MDR	523 (53.7%)	Y	–	Y	–	Y	Y	Y	Y	–	Y	Y	–	–	–	–
Conventional MDR TB regimen	MDR	139 (14.3%)	–	–	Y	Y	–	Y	Y	Y	–	–	Y	–	Y	–	–
Modified MDR/RR TB and FQ or SLI resistance regimen	MDR	31 (3.2%)	–	–	Y	Y	–	Y	Y	Y	–	Y	Y	–	Y	–	–
XDR TB regimen	XDR	26 (2.7%)	–	–	Y	Y	Y	Y	Y	Y	–	Y	Y	–	–	Y	Y
Modified for mixed pattern resistance regimen	DR	5 (0.5%)	–	–	Y	Y	–	Y	Y	Y	–	Y	Y	–	Y	Y	Y
New drug MDR TB and FQ/SLI resistance regimen	MDR	14 (1.4%)	–	–	Y	–	Y	–	Y	Y	–	Y	Y	–	Y	–	Y
New XDR TB regimen	XDR	10 (1.0%)	–	–	Y	Y	Y	–	Y	Y	–	Y	Y	–	–	–	Y
New MDR TB failure regimen	MDR	3 (0.3%)	–	–	–	–	Y	Y	Y	Y	–	Y	Y	–	–	–	–
New XDR TB failure regimen	XDR	2 (0.2%)	–	–	–	–	Y	Y	Y	Y	–	Y	Y	–	–	–	–
Shorter BDQ regimen	MDR	19 (2.0%)	–	–	Y	Y	–	Y	Y	Y	Y	Y	Y	Y	Y	–	–

Abbreviations: TB – tuberculosis, DR – drug-resistant TB, mono – single resistance to isoniazid, or other first-line drug (not rifampin), MDR – multi drug-resistant (i.e., resistance ≥2 first-line drugs but not to both isoniazid and rifampin), XDR – extensively drug-resistant (i.e., MDR and resistance to any fluoroquinolone and a second-line injectable drug), RR – rifampin-resistant, SLI – second-line injectable, H – isoniazid, FQ – fluoroquinolone, LFX – levofloxacin, R – rifampin, Z – pyrazinamide, E – ethambutol, BDQ – bedaquiline, MFX – moxifloxacin, ETO – ethionamide, CFZ – clofazimine, LZD – linezolid, CS – cycloserine, AM – amikacin, KM – kanamycin, PAS – para-aminosalicylic acid, CM – capreomycin

ETBD-F1 Adverse drug reaction (ADR) screening and referral form

(Fill in triplicate with carbon paper in sheets. Send one copy to the unit where patient is referred, give one copy to patient, and retain one copy for records)

Patient ID : ……………. **Month (number)** ___ **Date of screening**: _/_ /_

Mode of patient contact: Phone ☐ Home visit ☐ Hospital visit ☐

1	Headache सर दर्द’ ☐	9	Visual disturbances द्रष्टि का कम होना/ द्रष्टि मे बाधा ☐	17	Constipation कब्ज ☐
2	Dizziness चक्कर आना ☐	10	Heart pounding छाती में धड़धड होना ☐	18	Joint pain जोडो मे दर्द ☐
3	Sleep disturbances नींद में परेशानियाँ ☐	11	Nausea/Vomiting जी मचलाना/ उलटी होना ☐	19	Fatigue थकावट ☐
4	Seizures अकड़ी ☐	12	Loss of appetite भूख ना लगना ☐	20	Skin rash/itching त्वचा पे लाल चटके/हीव्स/खुजली ☐
5	Psychosis मनोविकृति ☐	13	Abdominal pain पेट में दर्द ☐	21	Yellowish discoloration of skin and eyes त्वचा और आँखों का पीलापन होना ☐
6	Diminished/lost hearing कम सुनाई देना/ सुनाई नहीं देना ☐	14	Diarrhoea दस्त ☐	22	Neck/ face swelling गर्दन में सुजन/ चहरे में सुजन होना ☐
7	Ringing in ears कानो का बजना ☐	15	Difficulty in urination मूत्र होने में कठिनाई होना ☐	23	Change in behaviour व्यवहार में बदलाव ☐
8	Depression/Suicidal thoughts डिप्रेसन/ आत्महत्या का विचार ☐	16	Tingling/pain/numbness in hands or feet झुनझुनाहट होना/ दर्द होना/ हाथ व पांव का सुन होना ☐	24	Other अन्य ☐
					**No adverse drug reaction** ☐

ADR number	Severity* 1.Severe 2.Not severe	Date of onset	Duration	Frequency/episodes (# per day)	Action taken 1.Not referred 2.Referred for ADR management	Remarks

* Severe: Life-threatening condition, requires inpatient hospitalization, resulting in disability or incapacity or death.

Name of health worker ______________________ Designation____________
Date of screening______________ Sign_____________

If referred for AE management

Name/address of health institution to which patient is referred ______________________________________
Date referred: _/_/_

………

For use of the health institution where the patient has been referred

Patient ID__________ Date of referral _/__
The above case reported to this health institution on______ for _____________at OPD/IPD.

Brief discharge summary (Diagnosis/Investigations/Treatment/follow-up/Hospitalized)
____
Diagnosis:
Action: Drug stopped ☐ Dose reduced ☐ Not changed ☐ Hospitalized: Yes/No
Outcome: Fatal ☐ Recovering ☐ No change ☐ Recovered ☐ Unknown ☐ Other ☐ (specify)____
Medical Officer/Physician_______________________________ Date_/_/__ sign__________
(Send this slip back to the referring health institution/facility)

Appendix Figure. "*End DRTB in Dharavi slum*" adverse event screening form and referral form.
